# Real-time observation of nucleation and growth of Au on CdSe quantum dot templates

**DOI:** 10.1038/s41598-021-97485-z

**Published:** 2021-09-21

**Authors:** Neelima Paul, Junyu Huang, Chang Liu, Taidong Lin, Chenghao Ouyang, Zhaoyu Liu, Chunfeng Chen, Zhongyi Chen, Zhiyin Weng, Matthias Schwartzkopf, Stephan V. Roth, Peter Müller-Buschbaum, Amitesh Paul

**Affiliations:** 1 Technische Universität München, Heinz Maier-Leibnitz Zentrum (MLZ), Lichtenbergstr. 1, 85748 Garching, Germany; 2 Guangdong Technion-Israel Institute of Technology, Shantou, 515063 Guangdong China; 3 Deutsches Elektronen-Synchrotron DESY, Notkestr. 85, 22603 Hamburg, Germany; 4 Department of Fiber and Polymer Technology, KTH Royal Institute of Technology, Teknikringen 56-58, 100 44 Stockholm, Sweden; 5 Physik-Department, Lehrstuhl für Funktionelle Materialien, Technische Universität München, James-Franck-Str. 1, 85748 Garching, Germany

**Keywords:** Materials science, Nanoscale materials, Nanoparticles, Nanoscience and technology, Nanoscale materials, Quantum dots

## Abstract

Semiconductor quantum dot (QD) arrays can be useful for optical devices such as lasers, solar cells and light-emitting diodes. As the size distribution influences the band-gap, it is worthwhile to investigate QDs prepared using different solvents because each of them could influence the overall morphology differently, depending on the ligand network around individual QDs. Here, we follow the nucleation and growth of gold (Au) on CdSe QD arrays to investigate the influence of surface ligands and thereby realized interparticle distance between QDs on Au growth behaviour. We particularly emphasize on the monolayer stage as the Au decoration on individual QDs is expected at this stage. Therefore, we sputter-deposit Au on each QD array to investigate the morphological evolution in real-time using time-resolved grazing-incidence small-angle X-ray scattering (GISAXS). The growth kinetics - independent of the template - signifies that the observed template-mediated nucleation is limited only to the very first few monolayers. Delicate changes in the Au growth morphology are seen in the immediate steps following the initial replicated decoration of the QD arrays. This is followed by a subsequent clustering and finally a complete Au coverage of the QD arrays.

## Introduction

Semiconductor quantum dots (QDs) expand the range of materials suitable for absorbers in solar cells due to their tunable bandgap^1^. This makes QD layers of highly absorbing semiconductors such as CdSe, CdTe, PbS, PbSe attractive options for solar cells in the nano dimension for solar energy conversion because their band gaps can be tuned or enhanced by QDs^2^. In case of PbS or PbSe based QD solar cells, this quantum size effect has been shown to cause an increase of the absorption edge of PbS or PbSe and that is useful towards preparation of QD based solar cells with high efficiencies^3–5^.

CdSe QDs are chemically stable and highly luminescent in the entire visible light range^6^. Moreover, electrons within dots occupy quantized energy levels and the size of the dots determines their degree of confinement and influences the occupied density of states affecting electrical and optical properties^7^. Therefore, several CdSe-based QD solar cells have been fabricated during the recent years^8–10^. Besides use in solar cells, other optical applications of such quantum dots are in lasers, light emitting diodes and quantum computation^3^. Size dependence of semiconducting QDs with tunable band-gap affect their optical properties. A sharp size distribution and a long-range order of QDs is beneficial, because it leads to a more uniform emission spectrum^11^. The photoluminescence can be further enhanced and the emission spectrum be broadened by combining these semiconductor QD with metals, such as Au or Ag, having plasmonic properties. Au growth on active semiconducting materials are important for their metallic contacts and or as charge recombination centers^12^. Therefore, it is a suitable material for overgrowth and subsequent monitoring of correlation effects. However, information on order and correlation effects, which is desirable for many device applications^13^, are only rarely available. Systematic monitoring of self-assembled growth behavior and corresponding morphological evolution of Au on QD array can help in engineering the design for optoelectronic devices with improved electrical properties.

Colloidal QDs are typically synthesized in a solution based synthesis method using passivating or stabilizing ligands (organic molecules) like Trioctylphosphine (TOP) and oleic acid (OA) to avoid their agglomeration. Thereafter, an exchange of these ligands with long chains is performed with those with shorter chains or by linker molecules to overcome transport limitations. Specific surface groups are maintained after each washing or exchange step^14^. Thus such colloidal QDs are composed of inorganic core (e.g. CdSe) and different organic shells, depending on the surface ligands. For the CdSe QDs used in this work, Photoluminescence (PL), UV-vis absorption spectroscopy, spectral and time dependant surface photovoltage (SPV) data on these QDs in different ligands ot linker molecules show that the surface of QDs has a high influence on PL signal and charge separation in CdSe QD thin films^15^.

Previously, we reported the mechanism of Au nanostructure nucleation and growth on CdSe QD arrays for toluene solvent as we probed the templating possibilities^16^. Au with a sufficient chemical contrast to CdSe, provides a large electron scattering density difference at the interface between cluster and the surrounding medium, thus making it suitable to monitor the nucleation and growth behavior. In this study, we investigate the role of solvents on the morphological evolution of the Au electrode layer on top of QD arrays, which have been formed using different passivating ligands. As these ligands influence the surface of the QDs more, they have a direct impact on the charge separation^17^. The impact of the two different solvents (*chlorobenzene*, *pyridine*) and a linker-molecule (*dithiol*) treatment is expected to affect the inter-QD distance, the surface as well as the area surrounding the QDs. In a previous study, similar QD layers were deposited by dip coating on ITO substrates and it was demonstrated that the distance between the QDs decreased after washing and ligand exchange when going from *chlorobenzene* to *dithiol*. Moreover, modulated SPV signals were strongly increased and also SPV transients became longer with successive ligand exchange^14^. The same group also performed UV–Vis absorption studies and reported differences in the absorption spectra and slight shifts in the first excitonic peak maximum position on ligand exchange and they attributed it to changes in dielectric environment^15^.

Thus, using solvents one can influence parameters such as (1) the interparticle distance (center-to-center) between neighbouring QDs, (2) the dielectric surroundings and (3) the electronic states on the surface of QDs. The first and the second factors strongly influence the absorption of light while the third factor mainly changes the charge carrier dynamics of excited electrons and holes. The thickness of the organic shell determines the packing of the QDs and therefore the fraction of CdSe in the layer. Irrespective of the solvent used, the diameter of QDs, measured from transmission electron microscopy (TEM) images is 4.5 ± 0.5 nm^18^. However, the micrographs reveal changes in the interparticle distance depending on the solvent used. The QDs arrays dispersed in *toluene* have a regular hexagonal arrangement and an interparticle distance of 7.7 ± 0.2 nm between centers of QDs. For QDs dispersed in *chlorobenzene*, the distance reduced to 6.4 ± 0.2 nm and the hexagonal order is maintained. For CdSe QDs dispersed in *dithiol*, the distance reduces to 5.5 ± 0.4 nm. In the case of CdSe QD layer dispersed in *pyridine*, the distance is similar to that of *dithiol* 5.9 ± 0.3 nm, and a loss of hexagonal order is seen^15^.

Thus, the average interparticle distance within the assemblies decreases from 7.7 nm (*toluene*) to 5.5 nm (*dithiol/pyridine*) while maintaining a similar QD diameter. The mechanism by which solar energy is harvested and transported to an interface for exciton dissociation within natural and artificial photovoltaic and photocatalytic systems is excitonic energy transfer (*EnT*)^19^. For energy harvesting applications, the rate of interparticle *EnT* is the key figure of merit. For a minimum distance, it must be fast enough to compete with the excitonic lifetime of the QD. Therefore, the important parameter is a precise knowledge of the distance-dependence of the rate and yield of *EnT* within the dipole–dipole approximation embedded in the rate constant $\mathcal {K}$ of the Förster equation

1 $$\begin{aligned} \mathcal {K}_{EnT} = \frac{2\pi }{\hbar } \mid J _{AB} \mid ^{2} \mathcal {D}_{EnT} \end{aligned}$$

$$\begin{aligned} \text {where}~J _{AB} = \frac{1}{4\pi \epsilon _{0}} \frac{\hat{r}_A\cdot \hat{r}_B-3(\hat{r}_A\cdot \hat{R})(\hat{r}_B\cdot \hat{R})\mid \mu _A\mid \mid \mu _B\mid }{R^{3}}. \end{aligned}$$

The terms $\mu _A$ and $\mu _B$ are the magnitudes of the transition dipoles of two QDs separated by a distance *R* and $\mathcal {D}$ is the combined density of states for *EnT*. The unit vectors connecting the centers of the QDs and the unit vectors of the transition dipoles are $\hat{R}$ and $\hat{r}_{i}$, respectively. Here, we have succeeded in reducing the interparticle distance of the QDs for different solvents and thereby monitoring subsequent evolution of surface morphologies during the Au deposition, which becomes significant for device engineering and dot-decoration aspects.

Real-time in situ Grazing incidence small-angle X-ray scattering (GISAXS) experiments^20,21^ are used to follow different stages of the Au nanomorphology evolution during the Au sputter deposition on the QD arrays prepared with the solvents *chlorobenze*, *pyridine* and linker-molecule *dithiol*. Recently, in situ GISAXS was used for the observation of Au sputter deposition on PbS QD solids^22^. In the kinetics of Au layer growth, from the expected four stages, three stages were resolved: (1) initially formed small Au clusters merge into medium clusters under the influence of the QD template. (2) Large Au clusters form gradually and dominate the surface. (3) Vertical growth of the Au layer^22^. In our study, the purpose of using different solvents is to introduce changes in the site distributions or lateral correlation lengths of the CdSe dot arrays. Thus, observing the growth morphology of Au provides an insight for determining the kinetics of initial nucleation, which not only depends on the inter-dot distances but also influence subsequent clustering as they evolve towards the formation of compact layers.

## Results and discussions

### Pre and post Au-nanostructure growth

#### Two-dimensional GISAXS data

**Figure 1:**
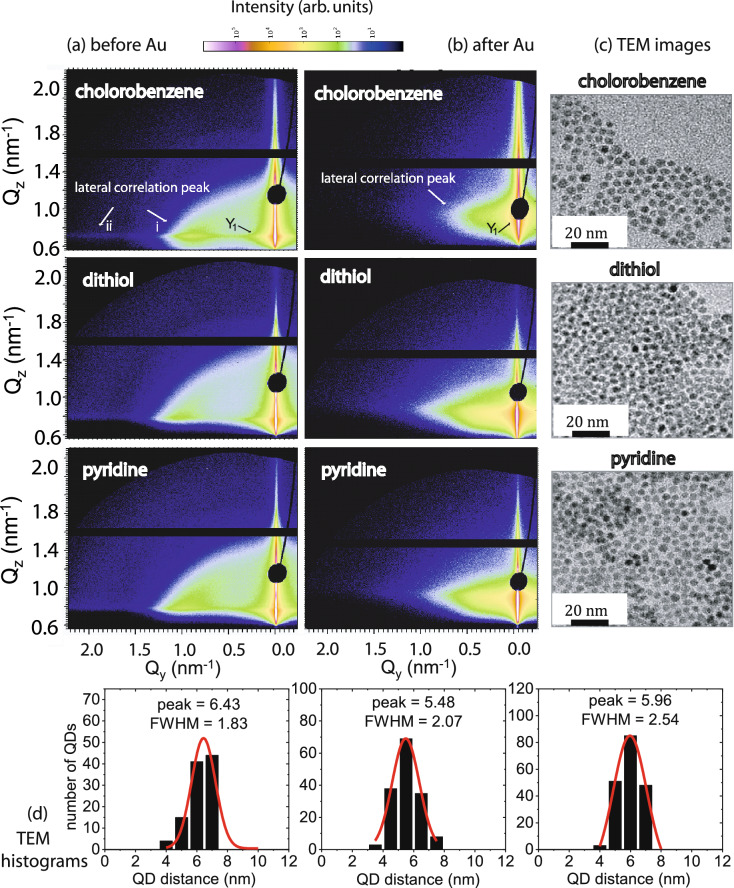
Two-dimensional GISAXS data of the QD array dispersed in three different solvents, namely *chlorobenzene*, *dithiol* and *pyridine* (**a**) before and (**b**) after Au deposition. The circular black shield due to the Pb beam stop is mounted to prevent detector saturation from the intense specular beam. The horizontal black line is the inter-module gap of the detector. (**c**) Corresponding TEM images of the bare QD templates are shown alongside. Reproduced from E. Zillner, *PhD thesis*, FUB,ISSN 1868-5781, page 55, by courtesy of Zillner^14^. (**d**) Size histograms from analysis of the TEM images. According to the imaging statistics, the mean interparticle distances of 6.4 ± 0.2 nm (*chlorobenzene*), 5.5 ± 0.4 nm (*dithiol*) and 5.9 ± 0.3 nm (*pyridine*) are estimated from the Gaussian fittings of the size distribution diagrams.

Figure 1a shows two-dimensional GISAXS data of the bare QD array, before Au deposition, whereas Fig. 1b shows the data after Au has been deposited on the array for 600 s. In the top row, some pronounced features such as Yoneda peak (*Y*), and side peaks due to lateral correlations are highlighted. The side peaks or prominent intensities at a certain localized position along the $Q_{\mathrm{y}}$, at the same $Q_{\mathrm{z}}$ positions as the Yoneda peak, are indicators of a very-well defined lateral structural order. The material characteristic Yoneda peak ($Y$ $_{1}$), causing a maximum in the scattered intensity, is located below the specular peak at $Q_{\mathrm{y}}$ = 0. Before Au deposition, the 1st and 2nd order lateral correlation peaks, indicated by the white arrows (i, ii), represent the in-plane nearest-neighbor and next-nearest-neighbor short-range order of QDs. They are visible at around $Q_{\mathrm{y}}$ = 0.92 nm$^{-1}$ (i) and 1.8 nm$^{-1}$ (ii) for *chlorobenzene* and respectively at around $Q_{\mathrm{y}}$ = 1.1 nm$^{-1}$ (i) and 2.1 nm$^{-1}$ (ii) for *dithiol* and at around $Q_{\mathrm{y}}$ = 1.0 nm$^{-1}$ (i) and 2.0 nm$^{-1}$ (ii) for *pyridine*. The estimated height of the QDs, using a spherical model, was estimated around 5 nm^16^. Following the 2D images of the bare QDs alone, we note that the lateral correlation peak in *dithiol* and *pyridine* has shifted to higher $Q_{\mathrm{y}}$ values corresponding to reduced interparticle spacing, as also confirmed by the corresponding TEM images (Fig. 1c). Size histograms compiled from the TEM data are shown in Fig. 1d. Analysis of the Gaussian fits gave an estimate of the mean interparticle distances. The shifts in the positions of correlation peaks in the GISAXS data are ascribed to changes in thickness of organic shells due to the different solvent treatments. After Au deposition, the long-range order distribution of the QDs is seen to be non-replicative. The different Au morphology are owed to the different distributions of their underlying dot-arrays.

#### Morphology evolution perpendicular to sample plane: before and after Au deposition

**Figure 2:**
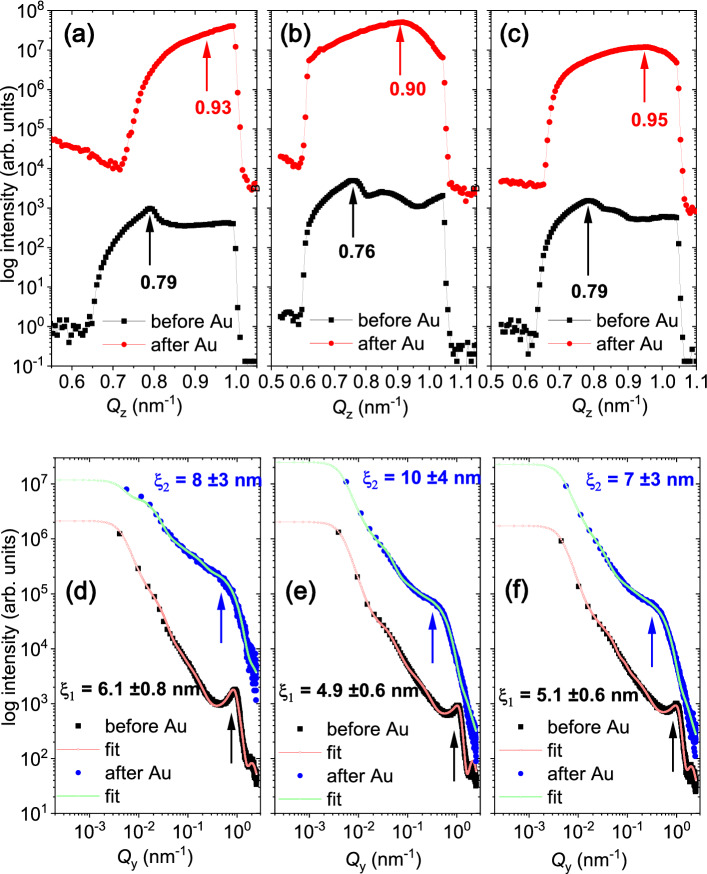
(**a**–**c**) One-dimensional vertical line cuts from the 2D GISAXS data at $Q_{\mathrm{y}}$ = 0 nm$^{-1}$ for a selected $Q_{\mathrm{z}}$ range measured for QD arrays prepared with different solvents namely (**a**) *chlorobenzene*, (**b**) *dithiol* and (**c**) *pyridine*. Two different Au thicknesses, $d_{\mathrm{Au}}$ = 0 nm and 14.4 nm are compared. (**d**–**f**) Respective one-dimensional horizontal line cuts along $Q_{\mathrm{y}}$ at $Q_{\mathrm{z}}$ = 0.79, 0.76 and 0.79 nm$^{-1}$ (before Au deposition) and $Q_{\mathrm{z}}$ $\approxeq$ 0.93, 0.9 and 0.95 nm$^{-1}$ (after Au deposition), representing the three different solvents. The horizontal line cuts are plotted along with their fits. The arrows indicate the characteristic vertical and lateral correlation peaks.

One-dimensional vertical line cuts from the two-dimensional GISAXS data along $Q_{\mathrm{z}}$ at $Q_{\mathrm{y}}$ = 0.0 nm$^{-1}$ are shown in Fig. 2a–c for the two solvents and a linker-molecule before and after Au deposition. The line cuts exhibit the Yoneda maximum ($Y$ $_{before}$), which corresponds to the experimentally observed critical angle $\alpha _{c}$ values (*Y* = $\alpha _{\mathrm{f}}$ + $\alpha _{c}$) before Au deposition and are given in Table 1 for the different solvents. The $Y$ $_{before}$ value 0.75 nm$^{-1}$ in the case of *toluene*^16^, which corresponded to an experimental scattering length density (SLD) of 2.44 $\times$ 10$^{-5}$ Å$^{-2}$, was similar to the theoretical X-ray SLD of SiO$_2$ (2.27 $\times$ 10$^{-5}$ Å$^{-2}$) substrates for the QD arrays. This $Y$ $_{before}$ value differs from the case of *dithiol* (0.76 nm$^{-1}$ leading to SLD = 2.73 $\times$ 10$^{-5}$ Å$^{-2}$) and also from *chlorobenzene* and *pyridine* (0.79 nm$^{-1}$ leading to SLD = 3.68 $\times$ 10$^{-5}$ Å$^{-2}$). All these estimated SLD values are obviously lower than that of CdSe (4.2 $\times$ 10$^{-5}$ Å$^{-2}$). It may also be noted that the average SLD on a large scale (relevant for the cuts at $Q_{\mathrm{y}}$ = 0.0 nm$^{-1}$) will be strongly lowered by the un-coated areas seen in the TEM images (Fig. 1c), which explains the average lower SLD values of the QD arrays. The differences in the SLD values originate from the differences in coverage and packing density, which is also supported by a more dense network of QDs for *dithiol* and *pyridine* as compared to *chlorobenzene*, seen in the TEM images. One may also note that the interparticle distances for both (*dithiol* and *pyridine*) are lower than the other two (*toluene* and *chlorobenzene*) by $\approx {40\%}$, which would affect the average SLD value due to increased packing density of QDs. As mentioned earlier, the thickness of the organic shell determines the packing density of the QDs and thereby the fraction of CdSe in the layer.

Post Au deposition, different modulations of the lateral and vertical intensity profiles give rise to a broader lateral correlation peak and a shift towards a significantly higher $Q_{\mathrm{z}}$ value ($Y$ $_{after}$ $\approxeq$ 0.9–0.95 nm$^{-1}$), which corresponds to a mixed layer of Au-CdSe (8.4–11.2 $\times$ 10$^{-5}$ Å$^{-2}$). One may note that the SLD of Au (12.46 $\times$ 10$^{-5}$ Å$^{-2}$) is significantly higher than that of CdSe (4.2 $\times$ 10$^{-5}$ Å$^{-2}$). Thus the increase in SLD value was tracked by the shift in the Yoneda peak position, which is a direct measure of the Au deposits upon sputtering.

#### Morphology evolution parallel to sample plane: before and after Au deposition

One-dimensional horizontal line cuts along $Q_{\mathrm{y}}$ at a certain $Q_{\mathrm{z}}$ value, corresponding to the respective Yoneda peak positions (Y), shown in Fig. 2d–f, are taken from the two-dimensional GISAXS data for different solvents and linker-molecule before and after Au deposition and are plotted in a log-log scale. The line cuts are analysed via fits, which are also shown in the figure. The QD arrays show a well-defined differences in the distribution of the QDs caused by the ligand exchanges. The nearest-neighbour order peak is marked by an arrow in Fig. 2d–f before Au sputtering and a next-nearest-neighbour short range ordering is also noticeable. Moreover, a broad hump from non-prominent structural order with larger correlation distances can also be seen. After Au deposition, the prominent nearest-neighbour order peak shifts to lower Q values (also indicated by an arrow), indicating prominence of larger mean sizes after sputter deposition. The disappearance of the next-nearest-neighbour order peak is also observed indicating a loss in order after Au deposition.

#### Extraction of lateral sizes and inter-particle distances

The corresponding inter-particle (or inter-QD) distances $\xi _{before}$ and $\xi _{after}$, obtained respectively from the fits to the data before (using the parameter $\xi _1$) and after Au deposition (using the parameter $\xi _2$), are given in Table 1. The corresponding QD diameters change from $2R_{before}$ to $2R_{after}$, respectively before (using the parameter $R_1$) and after Au deposition (using the parameter $R_2$) are also given in Table 1. The fits to the data along $Q_{\mathrm{y}}$ are shown in Fig. 2d–f for the three different cases. One may note that $R_1$, $R_2$ and $\xi _1$, $\xi _2$ are the parameters describing the most prominent nanostructures in the fitting routine that corresponds to the QDs^23^ before and after Au deposition, respectively. These values are in good agreement with those obtained from the earlier TEM measurements^18^. Overall, one can see that $R_1$, $R_2$ remain similar, while the $\xi _1$, $\xi _2$ (or interparticle distance) change, depending on the surface ligand used. Therefore, it becomes important to note that the delicate changes in the growth morphology lie in the immediate steps following the initial replicated decoration of the QD arrays, which essentially signifies the imperativeness of the present in situ GISAXS study.

**Table 1:** One-dimensional vertical line cuts along $Q_{\mathrm{z}}$ at $Q_{\mathrm{y}}$ = 0.0 nm$^{-1}$ yielding the Yoneda maxima (Y$_{before}$) before and (Y$_{after}$) after Au deposition.

Solvents	Vertical line cuts		Horizontal line cuts	
	$Y_{before}$ (*before Au*) (nm$^{-1}$)	$Y_{after}$ (*after Au*) (nm$^{-1}$)	$R_1$/$\xi _1$ (*before Au*) (nm)	$R_2$/$\xi _2$ (*after Au*) (nm)
Chlorobenzene	0.79 ± 0.01	0.93 ± 0.02	2.4 ± 0.4/6.1 ± 0.8	2.2 ± 0.6/8.0 ± 3
Dithiol	0.76 ± 0.01	0.90 ± 0.01	2.4 ± 0.3/4.9 ± 0.6	2.3 ± 0.8/10.0 ± 4
Pyridine	0.79 ± 0.01	0.95 ± 0.01	2.4 ± 0.4/5.1 ± 0.6	2.3 ± 0.8/7.0 ± 3
Toluene^16^	0.75 ± 0.01	0.92 ± 0.01	2.7 ± 0.4/6.7 ± 0.6	2.7 ± 0.7/10.0 ± 2

The data are tabulated for different solvents. Fit parameters, $R_1$, $R_2$ and $\xi _1$, $\xi _2$, extracted from the one-dimensional horizontal line cuts along $Q_{\mathrm{y}}$ before and after Au deposition at certain $Q_{\mathrm{z}}$ values, corresponding to their respective Yoneda peak positions (Y$_{before}$, Y$_{after}$).

### Intermediate morphologies during Au-growth

#### Vertical and horizontal line cuts

**Figure 3:**
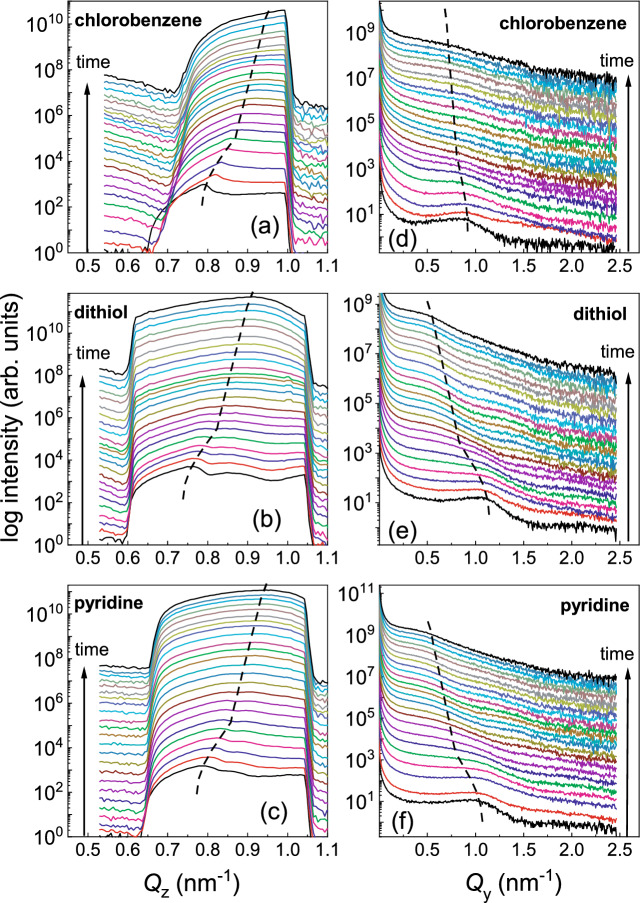
Temporal evolution of scattering patterns during the deposition of Au during 0–600 s corresponding to a Au thicknesses of $d_{\mathrm{Au}}$ = 0–14.4 nm on the QDs array. (**a**–**c**) One-dimensional vertical line cuts from the 2D GISAXS data at $Q_{\mathrm{y}}$ = 0 nm$^{-1}$ for a selected $Q_{\mathrm{z}}$ range measured for QD array prepared with different solvents namely, *chlorobenzene*, *dithiol* and *pyridine*. (**d**–**f**) Respective one-dimensional horizontal line cuts at constant $Q_{\mathrm{z}}$ values with three different solvents, namely *chlorobenzene*, *dithiol* and *pyridine*. The evolution of the vertical and lateral correlation peaks with deposition time are indicated by the dotted lines in each case. Out of the total number of frames ($\approx$ 6000) collected within 600 s, the data for the plots above were extracted for every 250th frame, which correspond to at an interval of 25 s and a thickness of around 0.6 nm.

Figure 3a–f shows twenty-four one-dimensional vertical line cuts along $Q_{\mathrm{z}}$ at $Q_{\mathrm{y}}$ = 0.0 nm$^{-1}$ and horizontal line cuts along $Q_{\mathrm{y}}$ at a certain $Q_{\mathrm{z}}$ value selected from the two-dimensional GISAXS data of Au sputter deposition on QD array, respectively. The line cuts cover a range from 0 to 600 s of deposition time for each of the three cases, which correspond to a Au film thicknesses $d_{\mathrm{Au}}$ = 0–14.4 nm.

The intensity of the *Y* peaks along the $Q_{\mathrm{z}}$ axis at $Q_{\mathrm{y}}$ = 0, are seen to be gradually shifting with deposition time from their initial positions at $Q_{\mathrm{z}}$ = 0.79 nm$^{-1}$ (*chlorobenzene*), 0.76 nm$^{-1}$ (*dithiol*) and 0.79 nm$^{-1}$ (*pyridine*) (Fig. 3a–c). These peaks in the plot of $Q_{\mathrm{z}}$ with time are the Yoneda maxima which are related to the critical angles of the substrate and the thin films above it. The apparent shift of the peaks along $Q_{\mathrm{z}}$ with time, is in fact a gradual evolution of three different convoluted $Q_{\mathrm{z}}$ maxima at different stages of time as and when one overshadows the other. The first peak ($\sim$ 0.75 nm$^{-1}$), which is prominent before deposition, is due to the SiO$_2$, as inferred earlier from the evaluated SLD value in Fig. 2a–c. The second peak, particularly for *dithiol* and *pyridine*, at around 0.86 nm$^{-1}$ can be attributed to a porous CdSe layer before the commencement of Au deposition. With an increase in $d_{\mathrm{Au}}$, this secondary peak eventually gets merged with a third peak ($\ge$ 0.9 nm$^{-1}$), as it keeps on shifting from 0.86 nm$^{-1}$ to higher $Q_{\mathrm{z}}$ values due to increasing percentage of Au within a mixed layer of CdSe-Au. After the Au deposition, only the third peak (at 0.9–0.95 nm$^{-1}$, depending upon the solvent used) is visible, indicating the complete coverage of the QD array following a mixed layer of CdSe-Au in all cases.

Before the onset of Au deposition, a prominent first order side peak from the CdSe QDs is seen in the plots of $Q_{\mathrm{y}}$ versus time for all three cases of surface ligands. The 1st order lateral correlation peaks at $Q_{\mathrm{y}}$ = 0.92 nm$^{-1}$ (*chlorobenzene*), 1.1 nm$^{-1}$ (*dithiol*) and 1.0 nm$^{-1}$ (*pyridine*) along the $Q_{\mathrm{y}}$ axis for different $Q_{\mathrm{z}}$ values (Fig. 3d–f), are also seen to be gradually shifting with the onset of Au deposition. A shift of the peak towards lower $Q_{\mathrm{y}}$ values with time is indicative of the evolution of lateral structures pertaining to larger sizes. Thus, a followup of the corresponding lateral structure correlations of different mixed layers at different stages of $d_{\mathrm{Au}}$ on top of the bare QDs is seen here.

#### Temporal evolution

##### Intensity maps

**Figure 4:**
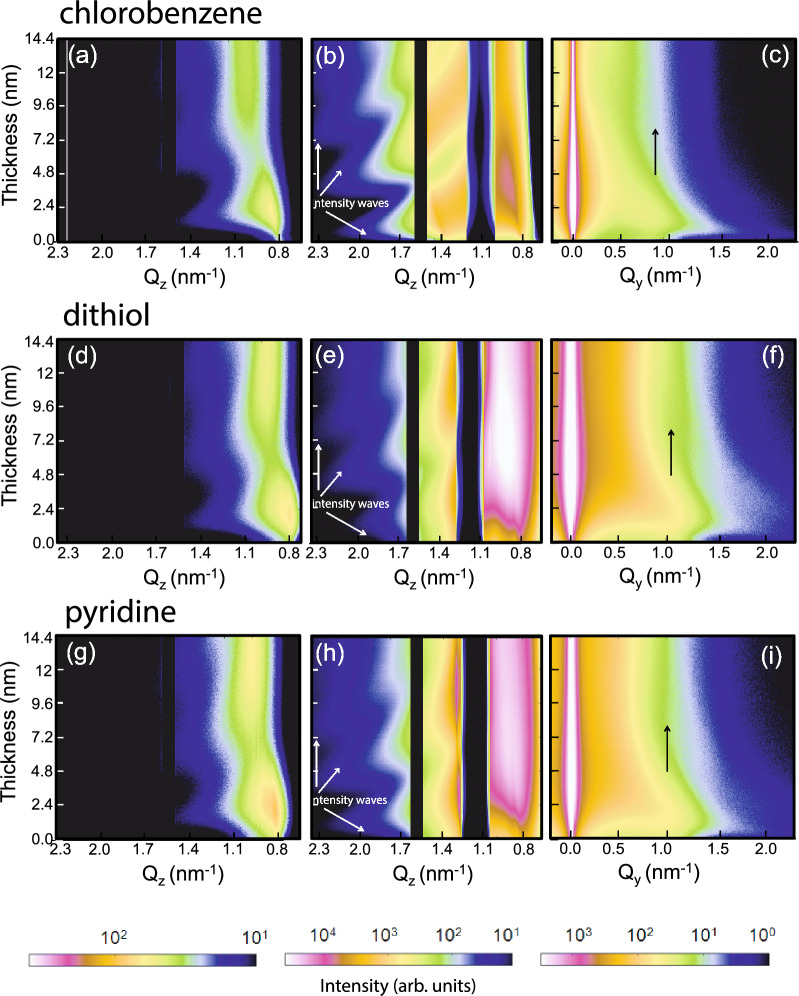
Time-resolved two-dimensional mapping extracts from vertical and horizontal line cuts of the GISAXS data taken at (**a**,**d**,**g**) $Q_{\mathrm{y}}$ = 0.92, 1.1 and 1.0 nm$^{-1}$ and at (**b**,**e**,**h**) $Q_{\mathrm{y}}$ = 0 nm$^{-1}$ for Au growth on QDs dispersed in different solvents, respectively for *chlorobenzene*, *dithiol* and *pyridine*. The diffuse scattering halo due to the nucleation and growth of the Au nanoclusters can be seen, the solid white arrows point out the resonant diffuse scattering characteristic of the growth signifying vertical correlation. The vertical black lines are owed to the inter-module gap of the detector. (**c**,**f**,**i**) Time-resolved two-dimensional GISAXS data showing the maps of horizontal line cuts taken at the respective Yoneda maximum positions $Q_{\mathrm{z}}$ = 0.79, 0.76 and 0.79 nm$^{-1}$ for the same samples. The solid black arrow follows the Au growth at QDs characteristic peak position due to lateral correlation.

Figure 4 maps the temporal evolution of the vertical and horizontal line cuts, concomitant with $d_{\mathrm{Au}}$, extracted from the two-dimensional GISAXS data. The vertical line cuts are taken at constant $Q_{\mathrm{y}}$ = 0.92, 1.1 and 1.0 nm$^{-1}$ (Fig. 4a,d,g) and at $Q_{\mathrm{y}}$ = 0 nm$^{-1}$ (Fig. 4b,e,h), respectively for the three QDs with different solvents and linker-molecule. The thickness evolutions give rise to the oscillating intensity waves or resonant diffuse scattering (RDS) in phase with the modulation of reflectivity with varying $Q_{\mathrm{z}}$ and can be seen more profoundly in Fig. 4b, being close to $Q_{\mathrm{y}}$ = 0 nm$^{-1}$. The widths of the profiles principally inform on vertical correlation along $Q_{\mathrm{z}}$ of the replicated in-plane structure, smaller than 2$\pi$/$Q_{\mathrm{y}}$ (where $Q_{\mathrm{y}}$ $\rightarrow$ 0). Thus, at higher $Q_{\mathrm{y}}$, such intensity modulations fade away. But they are still strong enough to infer upon the vertical correlation of in-plane structures of smaller frequencies.

Similarly, the maps of horizontal line cuts are taken at the respective Yoneda peak positions of the QDs dispersed in three different cases are shown in Fig. 4c,f,i. The peaks due to lateral correlation are intense in the initial stages. However, with subsequent deposition time or increasing $d_{\mathrm{Au}}$, the intensity gradually decreases as a consequence of cluster growth and coalescence. A better defined lateral structure (self-organization) is indicated by a lower FWHM of these peaks.

##### Off-centered cuts

**Figure 5:**
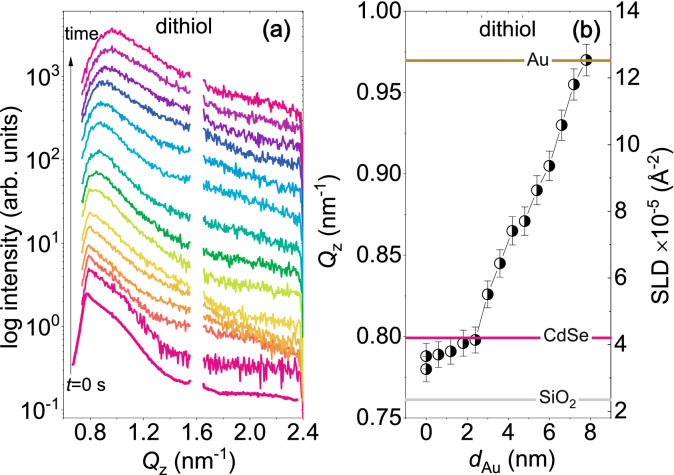
(**a**) One-dimensional vertical line cuts from the 2D GISAXS data at $Q_{\mathrm{y}}$ = 1.1 nm$^{-1}$ for a selected $Q_{\mathrm{z}}$ range measured for QD arrays prepared using *dithiol*. The Au thickness ranges from, $d_{\mathrm{Au}}$ = 0 nm to 8.0 nm for this data selection. (**b**) The shift in the $Q_{\mathrm{z}}$ values and the corresponding SLD values as a function of $d_{\mathrm{Au}}$.

The vertical line cuts along $Q_{\mathrm{z}}$ at $Q_{\mathrm{y}}$ = 0.0 nm$^{-1}$ indicate the vertical correlation along $Q_{\mathrm{z}}$ of the replicated in-plane structures, smaller than 2$\pi$/$Q_{\mathrm{y}}$. The intensity along $Q_{\mathrm{z}}$ is integrated over a rectangular spread of $\Delta Q_{\mathrm{y}}$ ± 0.04 nm$^{-1}$, the extent of which gives an estimated structural size of $\approx$ 160 nm. Thus, they will be dominated by unresolved larger scale structures, in particular defects, which can render much lower average SLD values. However, vertical line cuts taken at higher values of $Q_{\mathrm{y}}$ = 0.92 nm$^{-1}$ (*chlorobenzene*), 1.1 nm$^{-1}$ (*dithiol*) and 1.0 nm$^{-1}$ (*pyridine*), monitor the vertical correlation of smaller structures. Since these are the positions for the side maxima, they are representing the corresponding QDs, exclusively. Therefore, we plot the intensity of the vertical line cuts (along $Q_{\mathrm{z}}$) at the position of those higher $Q_{\mathrm{y}}$ values (1.1 nm$^{-1}$ for *dithiol*) as a function of time in Fig. 5a for *dithiol*, as an example, following their evolution at different stages of $d_{\mathrm{Au}}$.

Analysis of the line cuts are shown in Fig. 5b reveal that the calculated SLD values before sputter deposition corresponding to the peak along $Q_{\mathrm{z}}$ is 3.35 $\times$ 10$^{-5}$ Å$^{-2}$ for *dithiol*. One may recall that from similar vertical cuts at $Q_{\mathrm{y}}$ = 0.0 nm$^{-1}$, the SLD value is calculated as 2.73 $\times$ 10$^{-5}$ Å$^{-2}$ for *dithiol*, before sputter deposition. Thus, the SLD value for *dithiol* is closer to the SLD value of CdSe (4.2 $\times$ 10$^{-5}$ Å$^{-2}$) when measured at $Q_{\mathrm{y}}$, corresponding to the structure factor of the QDs. Thus, analysing the off-centered peaks provides a better estimate of the QD packing fraction as compared to that estimated from the centered (Y) peaks. During sputter deposition, the SLD values increase gradually with time until they attain a saturation level at round $d_{\mathrm{Au}}$ $\approx$ 8.0 nm, which matches the SLD value of Au (12.46 $\times$ 10$^{-5}$ Å$^{-2}$). Since it is challenging to follow the shift of $Q_{\mathrm{z}}$ with simultaneous shift of the peaks during the later stages towards lower $Q_{\mathrm{y}}$ values, we have restricted the evaluation up to the range where the SLD value reached to that of Au. An estimation of the FWHM of the peaks along $Q_{\mathrm{z}}$, rendering the extent of vertical correlation (2$\pi$/$\Delta Q_{\mathrm{z}}$), is seen to be around 30 nm, which is larger than the total Au thickness^24^. This signifies complete vertical correlation of vertically stacked interfaces or structures, which are of $\approx$ 6 nm (at $Q_{\mathrm{y}}$ $\sim$ 1.1 nm$^{-1}$) in size^25^. Signature of vertical correlation is also reflected in the RDS spectra (Fig. 4b,e,h).

#### Characteristic lateral structures

**Figure 6:**
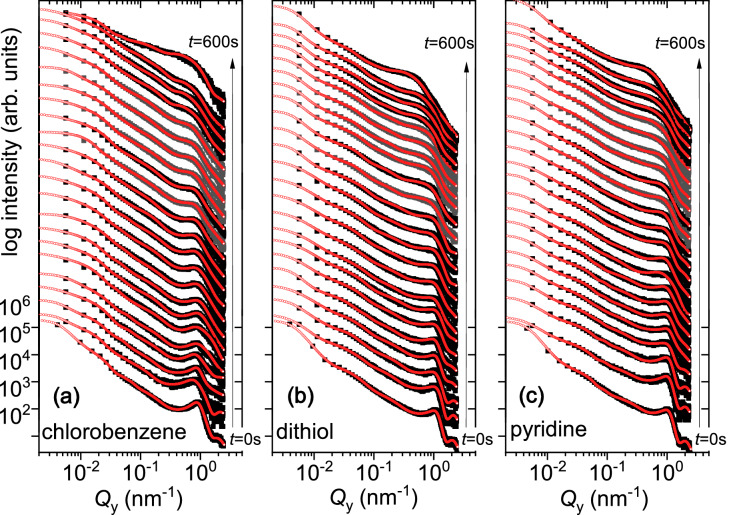
QDs prepared with (**a**) *chlorobenzene*, (**b**) *dithiol* and (**c**) *pyridine* solvents. Horizontal line cuts from the 2D GISAXS data along $Q_{\mathrm{y}}$ at different $Q_{\mathrm{z}}$ values along with their fits (red lines). They are plotted for different Au thicknesses from $d_{\mathrm{Au}}$ = 0 nm to 14.4 nm (from bottom to top).

In order to provide further support to our claim of reduced *R* and corresponding changes in $\xi$ values, we have plotted one-dimensional horizontal line cuts along $Q_{\mathrm{y}}$ at certain $Q_{\mathrm{z}}$ values in a log-log scale, corresponding to the respective Yoneda peak positions (Y) along with their fits within the LMA. The horizontal line cuts shown here, are for the three different cases of surface ligands *chlorobenzene*, *dithiol* and *pyridine* in Fig. 6a–c. They are depicting the nucleation and growth evolution as a function of $d_{\mathrm{Au}}$ or as increasing time of in situ sputter deposition from 0 to 600 s.

#### SLD values and structure evolution

**Figure 7:**
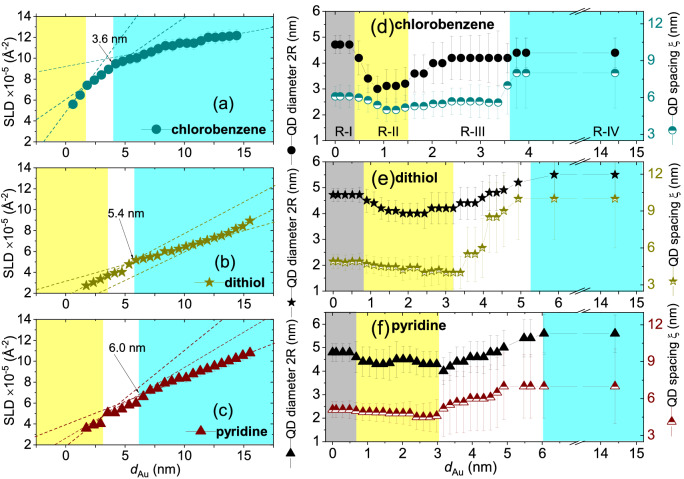
(**a**–**c**) Thickness dependence of SLD versus $d_{\mathrm{Au}}$ for the three solvents (**a**) *chlorobenzene*, (**b**) *dithiol* and (**c**) *pyridine* estimated from the one-dimensional vertical line cuts along $Q_{\mathrm{z}}$ at $Q_{\mathrm{y}}$ = 0 nm$^{-1}$. Out of total number of frames ($\approx$ 6000) collected within 600 s, the data for the plots above were extracted for every 250th frame, which correspond to an interval of 25 s and a thickness of around 0.6 nm. The three different slopes corresponding to different stages of real-time growth evolution of the species have been indicated. (**d**–**f**) Thickness dependence of the QD diameter (2*R*) and the corresponding inter-QD spacing ($\xi$) for the three solvents (**d**) *chlorobenzene*, (**e**) *dithiol* and (**f**) *pyridine*. Out of the total number of frames ($\approx$ 6000) collected within 600 secs, the data for the plots above were extracted for every 80 frames following the frame number 42–1642 (*chlorobenzene*), 54–2454 (*dithiol*) and 42–2522 (*pyridine*), which correspond to at an interval of 8 s and a thickness of around 0.2 nm. The overall growth morphology is divided into four different regions, R-I (gray region), R-II (yellow region), R-III (white region) and R-IV (cyan region).

The SLD values obtained from *Y* peaks for the three samples reveal that they are evolving with $d_{\mathrm{Au}}$ and are plotted in Fig. 7a–c. Initially, evolutions in SLD for each solvent show a linear increase with $d_{\mathrm{Au}}$. This linearity gets deviated beyond certain values of $d_{\mathrm{Au}}$ where it changes its slope. The changes in slopes occur at different *d*_Au_ values for different solvents *viz.*, $\approx$ 1.6 nm for *chlorobenzene*, $\approx$ 3.4 nm for *dithiol* and $\approx$ 3.2 nm for *pyridine*. An initial steeper slope usually indicates stronger vertical correlation, typical for a growth which maintains the preferred initial nucleation sites. Above a certain $d_{\mathrm{Au}}$, we observe a change or rather a decrease in the slopes. This change is at a similar Au thickness for solvents *dithiol* at $\approx$ 3.4 nm and at $\approx$ 3.2 nm for *pyridine*, but occurs at significantly different thickness for *chlorobenzene* corresponding to a Au thickness of $\approx$ 1.6 nm. The observed similarity between *dithiol* and *pyridine* could be due to the fact that both have similar initial dense coverage and inter-particle distance and thus the Au morphology evolves similarly. The change in slope could also be related to the appearance of additional nucleation sites for Au nanostructures, for example in the open areas between the QDs. Thereby the preferences of nucleation on top of the QDs gets gradually lost in consequence with the loss of the corresponding vertical correlation as are also evident from the broadening of the peaks. Broadening is evident from an increase in the FWHM along $Q_{\mathrm{z}}$, which can be seen in Fig. 3. With further increase in $d_{\mathrm{Au}}$, we find a second change in the slopes of SLDs for all three samples. The changes in slopes are again of similar values of Au thickness for *dithiol* at $\approx$ 5.4 nm and *pyridine* at $\approx$ 6.0 nm but at a quite different and lower Au thickness of $\approx$ 3.6 nm for *chlorobenzene*. These $d_{\mathrm{Au}}$ values indicate the beginning of the formation of a mixed CdSe-Au layer. Finally, we find another change in slope at slightly higher values of $d_{\mathrm{Au}}$ and SLDs (*viz.*, $\approx$ 7.2 nm for *chlorobenzene*, $\approx$ 11.8 nm for *dithiol* and $\approx$ 10.0 nm for *pyridine*), which are indicative of Au-dominated mixed layers of CdSe-Au.

After Au deposition, the QD dot arrays obviously attain the SLD values of mixed CdSe-Au, as has been observed earlier for *toluene*^16^, as an example. A mixed CdSe-Au layer, assuming a 50:50 composition, has an SLD value of 8.3 $\times$ 10$^{-5}$ Å$^{-2}$. The experimentally observed SLD values reached after 14.4 nm of Au deposition are $\approx$ 12.2 $\times$ 10$^{-5}$ Å$^{-2}$ for *chlorobenzene*, $\approx$ 8. 9 $\times$ 10$^{-5}$ Å$^{-2}$ for *dithiol* and $\approx$ 10.8 $\times$ 10$^{-5}$ Å$^{-2}$ for *pyridine*. Thus, the SLD values of the deposited layers for both *dithiol* and *pyridine* remains in the mixed phase of CdSe-Au while for *chlorobenzene* it moves much closer to pure Au (12.46 $\times$ 10$^{-5}$ Å$^{-2}$). This attainment of SLD$_{Au}$ is also indicated by the level of saturation attained in the SLD values for *chlorobenzene* in Fig. 7a as compared to the other two cases.

#### Form factor evolution with Au thickness

The plot in Fig. 7d–f shows the variation of the QD size (2*R*) and the inter-dot spacing ($\xi$) as a function of $d_{\mathrm{Au}}$. Note that the fitting parameters (2*R* and $\xi$) here, are varying from 2$R_{before}$ to 2$R_{after}$ and $\xi _{before}$ to $\xi _{after}$. One can divide their variations into four different regions of growth morphologies, R-I, R-II, R-III and R-IV. In R-I, one can observe no significant change in either 2*R* or $\xi$. In R-II, at around $d_{\mathrm{Au}}$ = 0.4 nm, 0.8 nm and 0.7 nm for *chlorobenzene*, *dithiol* and *pyridine*, respectively one can see a certain decrease in the values of 2*R*, while the values of $\xi$ remain fairly similar. Below these thicknesses (within R-I) both parameters remain fairly constant, which implies an initial preferential accumulation of Au on top of the QDs i.e., the Au nanostructures are perfectly decorating the QD arrays. Beyond these thicknesses (i.e., within R-II), the decrease in 2*R* is seen to continue up to around $d_{\mathrm{Au}}$ = 1.5 nm, 3.4 nm and 3.1 nm for *chlorobenzene*, *dithiol* and *pyridine*, respectively. Thus, the increasing Au height with time is affecting the growth morphology as they create a different cluster morphology deviating from the initial QD arrays. Above these thicknesses i.e., within R-III, however, both parameters start to increase gradually till they reach their respective saturation levels in corroboration with the respective change in slopes of their SLD values as seen in Fig. 7a–c. The region of saturation is labelled as R-IV, which occurs at certain values of $d_{\mathrm{Au}}$.

In this regard, one should note that the attainments of the SLD value of Au was shown earlier in Fig. 5b to be fulfilled for each of the solvents roughly around the same values of $d_{\mathrm{Au}}$ where we also obtain a saturation in $\xi$ (Fig. 7d–f). However, the evolutionary trend of the SLD values as obtained from the *Y* peak values in Fig. 7a–c do not match with the evolutionary trend as obtained from the vertical cuts at the positions of the side-peaks, shown in Fig. 5b. Since the estimated SLD values are for larger (at $Q_{\mathrm{z}}$ = 0.0 nm$^{-1}$ in Fig. 7a–c) and smaller (at $Q_{\mathrm{y}}$ $\ne$ 0.0 nm$^{-1}$ in Fig. 5b) in-plane structures, respectively, they obviously indicate different evolutionary effects as a function of $d_{\mathrm{Au}}$.

### Growth model

Dot-decoration of QD arrays were usually reported to undergo a random nucleation of Au nanostructures on the solid surfaces followed by lateral Au cluster accumulation, comprising of a smaller in-plane correlation length. As the growth proceeds and coalescence occurs, the in-plane correlation length increases^20,26,27^. Earlier using *toluene* as the solvent, an initial preferential accumulation of Au on top of the QD arrays was observed (dot-decorating). Above $d_{\mathrm{Au}}$ = 0.48 nm , no significant change in the dot diameter or the inter-dot spacing were observed, which continued up to $d_{\mathrm{Au}}$ = 2.5 nm. Above this thickness, the dot diameter was found to increase with a concomitant decrease in the inter-dot distance^16^.

**Figure 8:**
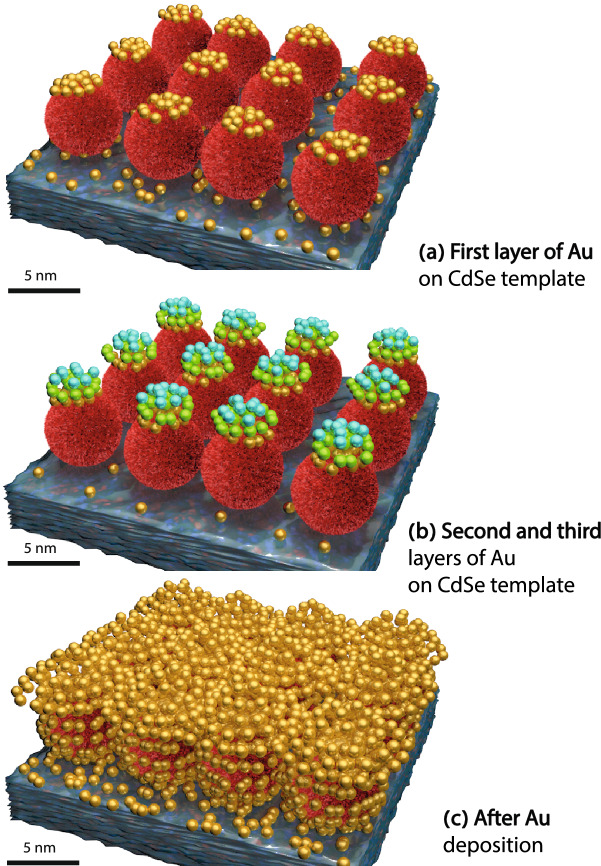
Diagrammatic sketch of Au nanostructure growth on QD arrays template depicting the case of *dithiol*, in particular: (**a**) template-mediated nucleation of Au on top of the QDs forming the first monolayer. (**b**) Dissociated *partial coverage of the nucleation site* and subsequent growth of Au nanostructures on top of the first monolayer. (**c**) Au coverage after a certain duration of in situ sputtering. We have used different shades for the Au on top of CdSe (red) than the adjacent ones for the first monolayer (golden) as also for the second monolayer (light green) and the third monolayer (cyan). This is to justifiably visualize the thickness dependent lateral correlation of the nanostructures.

In our present study, we find a different growth model. The morphologic evolution of Au nanostructure growth on the QD array is schematically depicted in Fig. 8. Initially, in R-I, there is an accumulation of Au atoms on top of the QDs (preferred nucleation sites) as we see no change in 2*R* or in $\xi$. This is the typical templating effect, which QDs array shows for deposited Au. Interestingly, the preferred decoration of dots is followed for the longest time for the *dithiol* linker-molecule corresponding to Au thickness of 0.8 nm and the shortest time for the *chlorobenzene* solvent corresponding to Au thickness of 0.4 nm. As mentioned previously, the solvents affect the surface and area surrounding the dots and thus the self-assembly growth of Au on QD dot arrays.

A gradual *decrease* in 2*R*, decreasing from the QD diameter of the template array, but without a significant variation in $\xi$ in R-II signifies a *partial coverage of the nucleation sites*. The reduction in the nanostructure diameter is maximum for *chlorobenzene* ($\approx$  30%), followed by *dithiol* ($\approx$  15%) and *pyridine* ($\approx$  5%). Note that this is unlike the growth morphology that was reported earlier for the *toluene* solvent^16^, where we did not observe such an early reduction of the nanostructural diameter. We believe that the difference can be related to the respective Cl, SH and N groups associated with the fundamental C–H group of the three surface ligands. The length of the ligands are guided by the associated groups attached to the fundamental group, which in turn controls the QD size distribution. Since *toluene* does not carry any such associated group, the nucleation centers provided by the CdSe template are more effective than the others. The identification of the brink of dissociation of the nanostructures from the underlying QD array is significant from the point of view of nucleation and growth of Au and also its dependence on the solvents used. One monolayer of *fcc* Au was calculated to posses a nearest neighbor distance of 0.283 nm^28^. Interestingly, the real-time GISAXS experiments helped us to find out that it is only the first two monolayers (for *chlorobenzene*), two to three monolayers (for *dithiol*) and more than two monolayers (for *pyridine*) of Au, which are adhering to the template-mediated nucleation. The third monolayer, grows on the partial nucleation sites, eventually to become completely independent of the QD array subsequently after a few (4–6) monolayers of Au more. Since the reduction in nanostructure diameter is higher in the case of *chlorobenzene*, the complete dissociation from the QD array is also relatively faster. In other words, it is more prone to grow upon a newly-found nucleation site. The partial coverage of nucleation site dependency continues as long as the lateral correlation order of the QD arrays is maintained, which therefore becomes an indicator of the growth evolution.

This morphology of partial coverage is eventually taken over by the usual coalescence of an accumulation of Au atoms at and around the QDs in R-III, as has been reported earlier in other studies^20,26^. Lateral Au growth around the QDs forming a mixed layer of CdSe-Au leads to a poor correlation order and larger nanostructural size. This coalescence is manifested by peak broadening and a shift of the characteristic scattering peak towards lower $Q_{\mathrm{y}}$ values, away from the initial QDs with a gradual *increase* in both 2*R* and $\xi$. This shift is found concomitant with the corresponding systematic formation of different phases of CdSe-Au nanostructures, respective to the three different surface ligands till they reach a saturation level in R-IV.

In R-IV, Au growth proceeds predominantly in the vertical direction where a complete layer is formed. At this stage of usual grain-growth, the templating effect of the original QDs is obviously lost.

Although each of these colloidal QDs arrays consists of the same inorganic core (CdSe), the QDs in their respective templates have different organic shells of molecules, depending on their treatment. Some have only organic molecules with long chains (such as TOP and OA), whereas others have shorter chains, or even linker molecules. During the Au deposition, Au molecules experience different organic environments around the QDs as they surface-diffuse on the QD template, which influences their nucleation and growth kinetics and are manifested by the different Au dot-decoration and eventual thickness on the QDs. Thus, we demonstrate that the Au growth behavior is extremely sensitive to any difference in the organic shells around the QDs arrays.

In summary, the nucleation and growth of Au on QDs mediated by surface ligands is manifested in the following way in the initial monolayer stage of growth. QDs arrays treated with *chlorobenzene* have Au metal atoms positioned directly on top of QDs for an Au thickness up to 0.4 nm whereas QDs arrays treated with *dithiol* have Au metal atoms positioned directly on top of QDs for double that Au thickness, i.e. up to 0.8 nm. For QD arrays treated with *pyridine*, results are similar to that for *dithiol* as there Au metal atoms are positioned directly on top of QDs for an Au thickness up to 0.7 nm.

## Conclusion

In this study, we investigate the role of solvents on the morphological evolution of the Au electrode layer on top of QD arrays, which have been formed using various passivating ligands. As these ligands influence the surface of the QDs, they have a direct impact on charge separation in semiconducting CdSe QD thin films. We chose CdSe QDs as our model system because they are quite stable in dispersion. The self-assembly of Au on each array is followed in real-time during their sputter growth by in situ GISAXS. In the initial growth phase, the highly mobile Au atoms prefer to nucleate and grow precisely on top of the QDs for about 0.4 nm of Au thickness for the QD array with *chlorobenzene* solvent. For the QD array with *dithiol* solvent, the dot-decoration thickness is about 0.8 nm of Au and for *pyridine* it is around 0.7 nm of Au thickness. The Au nanostructures initially follow the size of the QDs and then slightly decrease in size before finally forming a mixed Au-CdSe dot layer, and subsequently, the Au growth proceeds vertically to form an Au capping layer. By this stage, the templating effect is lost, and the morphology of the final layer corresponds to Au clusters larger in size as compared to the dots and with a larger size distribution. With this study, we demonstrated that those CdSe QD arrays, which are prepared with *dithiol* linker-molecule and show the improved modulated and transient SPV signals with respect to the pristine sample, also provide the best condition to have the Au metal nanocontacts positioned directly on top of QDs for a tailored and local contact formation with $d_{\mathrm{Au}}$ = 0.8 nm. Thereby, this is work offers useful insights towards engineering QD based photovotaic devices with superior optoelectronic properties.

## Methods

### Array synthesis

Array synthesis of the CdSe QDs was done by treating the QDs in different solvents and linker-molecule such as (a) * toluene* (b) *chlorobenzene* (c) *dithiol* and (d) *pyridine*. For preparation, the CdSe QDs were stabilized in (sub-)monolayers with (1) trioctylphosphine (TOP) and oleic acid (OA) and were subsequently dispersed in *toluene*. (2) The washed CdSe QDs (TOP + OA washed) were re-dispersed in *chlorobenzene*. (3) The exchange of ligands with 1,4-benzene-dithiol (*dithiol*) was done in a layer and not in dispersion. (4) The washed CdSe QDs were re-dispersed in *pyridine*. Details of the preparation of the QD arrays used in this study using various solvents or linker-molecule such as *toluene*, *chlorobenzene*, *dithiol*, *pyridine* can be found elsewhere^14^. Passivating ligands like TOP and OA were used in the synthesis to avoid agglomeration and preserve the confinement of charges. A thin layer of CdSe QDs was prepared on clean SiO$_{2}$ substrates by dip coating using a constant withdrawal speed of 1 mm/s under an inert atmosphere as described elsewhere in detail^14^. Here, we present the real-time growth mechanism of Au on CdSe QD arrays dispersed in *chlorobenzene* (C$_{6}$H$_{5}$Cl), *dithiol* (C$_{6}$H$_{4}$(SH)$_{2}$) and *pyridine* (C$_{5}$H$_{5}$N) and compare it with that dispersed in *toluene* (C$_{7}$H$_{8}$)^16^.

### In-situ GISAXS

The in situ time-resolved GISAXS sputter deposition experiments with Au target were performed at the Micro-and Nanofocus X-ray scattering (MiNaXS) beamline P03 of the synchrotron source with high brilliance at PETRA III in DESY, Hamburg^29^. For depositing Au, an ultra-high vacuum sputter deposition apparatus RF HASE with a 99.999% Au target (from MaTecK GmbH) was used^30^. Au atoms were sputtered perpendicular to the sample surface under a pressure of 15 $\upmu$bar for 600 secs in Ar gas atmosphere with an applied power of 100 W at room temperature. To have an estimation of the final Au film thickness, Au was also simultaneously deposited on a bare Si substrate for 600 s^16^. X-ray reflectivity fitting of this film had yielded Au film thickness of $d_{\mathrm{Au}}$ = 14.4 ± 1.7 nm, and rendered a nominal sputter deposition rate of 0.024 ± 0.003 nm s$^{-1}$.

A two-dimensional (2D) PILATUS 300 K detector (DECTRIS Ltd) was placed at a distance of 2011 mm from the sample position. With its readout time of 3.6 ms and the intense photon flux, it was possible to collect the scattered X-rays with high acquisition throughput during the sputter deposition. The selected wavelength was 0.09537 nm and the selected angle of incidence was 0.5° for all measurements. A point-like movable beam stop was placed just in front of the detector to block the specularly reflected beam which would otherwise have saturated the detector. The detailed experimental set-up is described in detail elsewhere^20^.

The momentum transfer vector $\vec {Q}$ can be written as its various components:

2 $$\begin{aligned} \vec{Q}=\vec{k_i} - \vec{k_f} \end{aligned}$$

$$\begin{aligned} \begin{pmatrix} Q_x\\ Q_y\\ Q_z \end{pmatrix} = k\begin{pmatrix} \cos \alpha _f\cos \chi -\cos \alpha _i\\ \cos \alpha _i\sin \chi \\ \sin \alpha _i+\sin \alpha _f \end{pmatrix} \end{aligned}$$

where $\alpha _i$ and $\alpha _f$ are incident and exit angles for scattering in the (*x*,*z*) plane, and $\chi$ is the scattering angle in the (*x*,*y*) plane. The sample is located in the (*x*,*y*) plane. The $Q_{\mathrm{x}}$ element can be ignored in the case of GISAXS geometry for modelling or analysis as we do not resolve the intensities (but integrate) along that axis^31–42^.

A two-dimensional (2D) intensity map was recorded after the X-ray beam had scattered from the sample. To the 1st order, the scattered intensity is proportional to the square of the particle form factor times the convolution product of the interference function^43–45^. The total scattering intensity is an incoherent sum of the intensities from each domain of monodisperse subsystems weighted according to the size-shape distribution and is given by

3 $$\begin{aligned} I(q) = \langle \mid F(q,R)\mid ^{2} * S(q,R)\rangle _{D} \end{aligned}$$

where * denotes the convolution product and $\langle \cdots \rangle _{D}$ is the average over coherent domain *D* enclosing the form factor $\mid$F(*q*,*R*)$\mid$ $^{2}$ in which the local interference function S(*q*,*R*) can depend on the particle size^46^. Form factors of form

4 $$\begin{aligned} \mid F(q,R,H)\mid ^{2} = \mid 2\pi R^{2}H \frac{\mathcal {J}_{1}(q_{\parallel }R)}{q_{\parallel }R} \sin _{c}(\frac{q_{z}H}{2})e^{i{q_{z}H}/{2}}\mid ^{2}, \end{aligned}$$

with cylindrical geometry distributed over a 1-D paracrystalline lattice, were considered^47^. Here, the cardinal sine $\sin _{c}$ = $\frac{\sin x}{x}$, $\mathcal {J}_1$ the Bessel function of first order, $q_{\parallel }$ = $\sqrt{q_{x}^{2}+q_{y}^{2}}$ and *H* is the cylinder height. The distribution of particles is given by the inter-particle correlation or local interference function S(*q*,*R*). The structure factor S(*q*,*R*), on omitting the homogeneous part, is expressed with the pair correlation function *g*(*R*) as

5 $$\begin{aligned} S(q) = 1 + n_{p} \int [g(R)-1]\exp [{{iq\cdot R}}] dR \end{aligned}$$

where n$_{p}$ is the number density of particles. The respective correlation function, apart from the structure factor contribution ($\exp [{-\frac{R}{\xi }}]$) accounting for the finite size effects^47^, is given by

6 $$\begin{aligned} S(q) = (1-\exp [-q^{2}\sigma ^{2}])/[(1+\exp [-q^{2}\sigma ^{2}]-2\exp [-\frac{1}{2}q^{2}\sigma ^{2}]\cos (q,\xi )] \end{aligned}$$

where $\sigma$ and $\xi$ are respectively the square-root of the variance and the mean value of the distance probability or the correlation length^46^.

Following the measurements, one-dimensional (1D) vertical (along $Q_{\mathrm{z}}$) and horizontal (along $Q_{\mathrm{y}}$) line cuts were extracted from the 2D maps. The horizontal line cuts were fitted with the software Genplot version v.2.11 (http://www.genplot.com/) by Computer Graphic Service Ltd to extract structural information in the lateral plane. For modelling the data, the Local Monodisperse Approximation (LMA) was applied, which is often used to describe a polydisperse system by separating the form factor from the interference function. Results of this fitting yielded average center-to-center distances ($\xi$), which were associated with the structure factors of the scattering objects comprising of three different cylindrical form factors with radii (*R*). The size distributions of radii ($\Delta R$) as well as the standard deviations of correlation lengths ($\sigma$) were used as additional fit parameters in the model.
